# The Pol β variant containing exon α is deficient in DNA polymerase but has full dRP lyase activity

**DOI:** 10.1038/s41598-019-45846-0

**Published:** 2019-07-09

**Authors:** Da-Peng Dai, Rajendra Prasad, Phyllis R. Strauss, Samuel H. Wilson

**Affiliations:** 1 Genome Integrity and Structural Biology Laboratory, National Institutes of Health, NIEHS, 111 T.W. Alexander Drive, P.O. Box 12233, Research Triangle Park, North Carolina, NC 27709 USA; 2 Present Address: The MOH Key Laboratory of Geriatrics, Beijing Hospital, National Center of Gerontology, Beijing, 100730 P.R. China; 3 Present Address: Biology Department, Northeastern University, 360 Huntington Avenue, Boston, MA 02115 USA

**Keywords:** Biochemistry, Molecular biology

## Abstract

DNA polymerase (Pol) β is a key enzyme in base excision repair (BER), an important repair system for maintaining genomic integrity. We previously reported the presence of a Pol β transcript containing exon α (105-nucleotide) in normal and colon cancer cell lines. The transcript carried an insertion between exons VI and VII and was predicted to encode a ~42 kDa variant of the wild-type 39 kDa enzyme. However, little is known about the biochemical properties of the exon α-containing Pol β (exon α Pol β) variant. Here, we first obtained evidence indicating expression of the 42 kDa exon α Pol β variant in mouse embryonic fibroblasts. The exon α Pol β variant was then overexpressed in *E. coli*, purified, and characterized for its biochemical properties. Kinetic studies of exon α Pol β revealed that it is deficient in DNA binding to gapped DNA, has strongly reduced polymerase activity and higher *K*m for dNTP during gap-filling. On the other hand, the 5′-dRP lyase activity of the exon α Pol β variant is similar to that of wild-type Pol β. These results indicate the exon α Pol β variant is base excision repair deficient, but does conduct 5′-trimming of a dRP group at the gap margin. Understanding the biological implications of this Pol β variant warrants further investigation.

## Introduction

DNA polymerase β is the smallest naturally occurring cellular DNA polymerase and is found as a single polypeptide chain of ~39 kDa with only 335 amino acid residues^1,2^. This enzyme is one of the most well-studied mammalian DNA polymerases, owing to its small size, stability upon recombinant expression and mono-subunit structure^3–6^. Pol β plays key gap-filling and gap trimming roles in base excision repair, both in the single-nucleotide or short-patch (SP) and long-pacth (LP) sub-pathways of BER^7–15^. Deletion of Pol β in mice leads to embryonic lethality or post-natal death^16,17^, indicating a required role of Pol β in development. Biochemical and structural studies revealed that Pol β consists of two main globular domains: The small N-terminal domain (8 kDa) possessing metal-independent dRP-lyase activity; and the larger C-terminal domain (31 kDa) carrying the metal-dependent DNA polymerase activity^6,18,19^. During SP BER, these domains are responsible for removal of the 5′-sugar phosphate group and template directed insertion of a dNMP into the DNA gap produced by AP endonuclease 1 (APE1) incision of the AP-site containing BER intermediate^7,8,15,19,20^.

After the cDNA cloning for human Pol β^4,21^, the genomic structure of the human Pol β gene was determined, revealing that it contains 14 exons and 13 introns spanning 33 kb^22^. Using the reverse transcriptase-polymerase chain (RT-PCR) assay, we and others found variant transcripts of the Pol β mRNA in human cell lines: A 58-bp deletion of exon II; and a 87-bp deletion of exon XI^22–24^. In addition to these splicing isoforms, we also observed a variant with a 107-bp insertion between exons IX and X, and this variant also was reported by another research group at the same time^25^. The exon II deletion and 107-nucleotide insertion have been suggested to be related to the development of colon cancer and Werner syndrome, respectively^25,26^.

To further characterize the splicing isoforms of Pol β in human cells, we previously performed RT-PCR analysis in several different human cancer and normal cell lines^27^. As a result, eight mRNA isoforms were detected among different cell lines. These isoforms contained various different types of deletions and/or insertions, most of which would cause frame shifts resulting in truncated polypeptides. An exception was the detection of an isoform containing a 105-nucleotide insertion. This 105-nt in-frame insertion, named exon α, is inserted at the junction site between exons VI and VII. Due to its in-frame coding properties, exon α is expected to be translated into 35 additional amino acid, leading to an enzyme variant of 42 kDa molecular mass. Little is known about the expression and function of this Pol β variant. Here we report that the recombinant form of the 42 kDa exon α Pol β variant was overexpressed in *E. coli*, purified and characterized for its BER properties.

## Materials and Methods

### RT-PCR analysis of exon α Pol β variant transcripts in cancer cells

Several different types of human cell lines were studied in the current work, including MCF7 (breast), AGS (stomach), A549 (lung) and Y79 (eye). These cell lines were from the ATCC and grown at 37 °C with 10% CO_2_ using culture medium suggested by the supplier. Total RNA was isolated using the RNeasy kit from Qiagen, and 1 μg RNA was used for generation of cDNA templates using the SuperScript III First-Strand Synthesis System (Invitrogen) in a final volume of 20 μl. Two microliter of cDNA template was then used for amplification from exon V to exon X with Forward primer 5′-TTCGGCAGGATGATACGA-3′ and Reverse primer 5′-TTGGCTGTTTGGTTGATT-3′. The sizes of amplicons for wild-type Pol β and exon α Pol β variant were 363-bp and 468-bp, respectively. To monitor the amount of template actually present in the reaction, the GAPDH expression level was also measured using primer pairs as follows: Forward primer, 5′-TGTTGCCATCAATGACCCCTT-3′; and Reverse primer, 5′-CTCCACGACGTACTCAGCG-3′. Amplification products were separated by electrophoresis in a 2.5% agarose gel and visualized by ethidium bromide staining. RT-PCR products were subsequently purified using the QIAquick Gel Extraction Kit (Qiagen) and confirmed by sequencing.

### Cloning, expression and purification of recombinant wild-type Pol β and exon α Pol β variant

The transcripts of full-length wild-type Pol β and 105-bp insert corresponding to the exon α Pol β variant were amplified from HeLa cells using primer pairs as follows: Forward primer, 5′-CTGCTCCCGTCTCCAAGT-3′; Reverse primer, 5′-CCAAAGACCCTTACATAGC-3′. Insertion of exon α was confirmed by DNA sequencing. Then, the open reading frame cDNAs of wild-type Pol β and the exon α Pol β variant were subcloned into Sal I and Not I sites in the pGEX-4T-3 (TEV) expression vector containing an amino-terminal GST-tag. The recombinant proteins were expressed in *E. coli* (BL21(DE3)-CodonPlus-RP) cells by cultivation in 2 liters of LB with ampicillin (100 μg/ml) and chloramphenicol (34 μg/ml) at 37 °C to an OD_595_ of 0.8–1.0; then, the temperature was lowered to 15 °C. Isopropyl-β-D-1-thiogalactopyranoside (IPTG) was added to a final concentration of 0.5 mM, after which expression was continued overnight at 15 °C. Cells were collected by centrifugation at 4,000 rpm for 30 min at 4 °C and stored at −80 °C until use.

The cell pellet was resuspended in 50 ml Buffer A containing 25 mM Tris, pH 7.5, 500 mM NaCl, 1 mM EDTA, 0.5 mM TECP, plus protease inhibitors (10 mM sodium metabisulfite, 1 mM 4-(2-aminoethyl)benzenesulfonylfluoride (AEBSF), 1 mM benzamidine, 1 μg/ml pepstatin A, and 1 μg/ml leupeptin). The cell suspension was sonicated and clarified by centrifugation at 166,080 × *g* for 30 min at 4 °C. Glutathione S-transferase (GST) Sepharose 4B (GE Healthcare, Bio-sciences, Pittsburg, PA) was prepared according to the manufacturer’s instructions, and washed twice with Buffer A containing 2 mM DTT to enhance binding capacity of GST-tagged Pol β. GST Sepharose 4B resin (~10 ml) was mixed with the supernatant fraction to bind GST-tagged Pol β in-batch and shaken on a rocking platform at 4 °C for 3–4 h. Then, the resin was collected by centrifugation at 1,000 rpm for 1 min at 4 °C and washed 4–5 times with Buffer A plus protease inhibitors, as above. The resin was then washed 4–5 times with 35 ml Buffer B (25 mM Tris, pH 7.5, 75 mM NaCl, 1 mM EDTA, 1 mM DTT plus protease inhibitors) and resuspended in ~14 ml Buffer B. The GST tag was removed by adding 1 ml Tobacco Etch Virus (TEV) protease (1 mg/ml) and then incubating the sample overnight at 4 °C on a rocking platform. The supernatant fraction after centrifugation at 1,000 rpm for 1 min at 4 °C was collected, and the resin was washed twice with 20 ml Buffer B. The washes were combined with the supernatant fraction. While the GST-tag remained attached to the resin, Pol β and the exon α Pol β variant, respectively, were released. Further purification of the enzymes was then performed.

The supernatant fractions and washes containing each enzyme were exchanged with Buffer C (25 mM MES, pH 6.5, 1 mM EDTA, 0.5 mM TECP) to adjust the pH. The samples containing wild-type Pol β or the exon α Pol β variant were applied to a RESOURCE S (6 ml) column, washed with Buffer B or Buffer C, and then eluted with a linear NaCl gradient (75 mM to 1 M) in Buffer B or Buffer C, respectively. Column fractions (3 ml) were collected, and an aliquot (10 μl) of each or an alternate fraction was analyzed using a Nu-PAGE 4–12% Bis-Tris mini-gel system and MES buffer. Fractions containing Pol β (~39 kDa) or the exon α Pol β variant (~42 kDa) were pooled, concentrated, exchanged into storage buffer (25 mM Tris-HCl, pH 7.5, 150 mM NaCl, 1 mM β-mercaptoethanol, 0.5 mm EDTA, and 20% glycerol), aliquoted (100 μl each), and stored at −80 °C. Protein concentration was determined using the Bio-Rad Protein Assay Kit (Hercules, CA).

### Cell extract preparation

Wild-type Pol β expressing and null MEF cells were grown at 37 °C in a 10% CO_2_ incubator in Dulbecco’s modified Eagle’s medium supplemented with L-glutamine (Invitrogen, Carlsbad, CA) and 10% fetal bovine serum (HyClone, Logan, UT). Cell extracts were prepared, as previously described^28^. Briefly, cells were washed twice with phosphate-buffered saline at room temperature, detached by scraping, collected by centrifugation and resuspended in Buffer I (10 mM Tris-HCl, pH 7.8, 200 mM KCl, and protease inhibitor cocktail). An equal volume of Buffer II (10 mM Tris-HCl, pH 7.8, 200 mM KCl, 2 mM EDTA, 40% glycerol, 0.2% Nonidet P-40, 2 mM dithiothreitol (DTT), and protease inhibitor cocktail) was added. The suspension was rotated for 1 h at 4 °C, and the resulting extract was clarified by centrifugation at 20,800 × *g* for 10 min at 4 °C. The protein concentration of the extract was determined by Bio-Rad protein assay using bovine serum albumin (BSA) as a standard.

### Immunoblotting analysis

Immunoblotting was performed essentially as described^29^. Briefly, whole cell extracts (20 μg) from wild-type Pol β expressing MEF and null MEF cells were separated by NuPAGE 4–12% Bis-Tris gel and transferred onto a nitrocellulose membrane. Purified wild-type Pol β (10 ng) and exon α Pol β were included in the gel lanes, as indicated. The membrane was blocked for 1 h at room temperature in 5% nonfat dry milk in Tris-buffered saline (TBS) containing 0.1% (v/v) Tween 20 (TBST). The membrane was incubated with polyclonal antibody raised in rabbits against the 8 kDa amino-terminal domain of Pol β (1:1000)^7^. After 2 h incubation at room temperature, the membrane was washed three times with TBST and incubated for 1 h with goat anti-rabbit IgG (H + L)-horseradish peroxidase (HRP) conjugate (1:10,000). The HRP activity was detected by an enhanced chemiluminescence system using SuperSignal West Pico Chemiluminescent substrate and Amersham Imager 600 (GE Healthcare)

### DNA substrates

A 34-bp DNA substrate containing a single-nucleotide gap was prepared by annealing three deoxyoligonucleotides, for the DNA binding assay and the single-turnover gap-filling assay, as follows: The annealing procedure was carried out by incubating a 5′-FAM labeled 15-mer primer and an 18-mer downstream oligonucleotide with a 34-mer complementary DNA strand, in an equimolar ratio, and then heating the solution at 90 °C for 3 min after which time the solution was allowed to slowly cool to 25 °C. Similarly, for the dRP lyase or NaBH_4_ cross-linking experiments, the duplex DNA (34-bp) containing a nick at positions 16 was prepared by annealing a 3′-FAM labeled 19-mer containing a 5′-phosphate and uracil and an upstream 15-mer DNA complementary to a 34-mer lower strand DNA. For the BER assay, the DNA substrate was prepared by annealing a 35-mer DNA with uracil at position 16 to a 35-mer complementary DNA.

### DNA binding estimate for wild-type Pol β and the exon α Pol β variant

Binding incubations were at room temperature in a total volume of 20 μl for 5 min. Different concentrations (0–64 nM) of wild-type Pol β or the exon α Pol β variant and 5′-FAM labeled 1-nt gapped DNA (10 nM) were mixed in a buffer containing 50 mM Tris-HCl, pH 7.5, 100 mM KCl, 5 mM MgCl_2_, 1 mM DTT, 10% glycerol, and 100 μg/ml BSA. After incubation, the enzyme-DNA complexes were separated from free DNA by 10% non-denaturing polyacrylamide gel electrophoresis at 4 °C. A Typhoon PhosphorImager (GE HealthCare) was used for scanning the gels.

### Gap-filling DNA synthesis

The DNA polymerase gap-filling assay under enzyme excess conditions was done at 37 °C in a final volume of 50 μl, as described previously^30^. The reaction mixture contained 50 mM Tris-HCl, pH 7.5, 100 mM KCl, 5 mM MgCl_2_, 1 mM DTT, 10% glycerol, 100 μg/ml BSA, 500 nM exon α Pol β, 50 nM gapped DNA substrate and various concentrations of dGTP (20, 100, 200, 500, 1000, 2000 µM). Samples corresponding to each dGTP concentration (5 μl each) were withdrawn at 30, 60, 120, 180, 240, and 300 s of incubation and quenched with 100 mM EDTA. Then, the samples were denatured, and products were separated by 16% denaturing PAGE in 89 mM Tris-HCl, pH 8.8, 89 mM boric acid, and 2 mM EDTA at room temperature. A Typhoon PhosphorImager (GE HealthCare) was used for scanning the gels and GraphPad Prism software (version 8.0) was used for data plotting. Measured activities were fitted to the Michaelis Menten equation by non-linear least-squares methods.

### UDG treatment of the DNA substrate

The 3′-FAM-labeled duplex oligonucleotide was treated with purified UDG. This resulted in the 3′-FAM-labeled DNA substrate with a 5′-dRP group at the nick. Typically, 400 nM DNA substrate was pretreated with 50 nM UDG in 50 mM HEPES, pH 7.4, 1 mM EDTA, and 2 mM dithiothreitol. The reaction mixture was incubated for 45 min at 30 °C, and kept on ice until use.

### dRP lyase assay

The dRP lyase activity was assayed essentially as described previously^31,32^. Briefly, the reaction mixture (10 µl) contained 50 mM HEPES, pH 7.5, 20 mM KCl, 2 mM dithiothreitol, 1 mM EDTA, and 200 nM UDG pretreated 3′-FAM-labeled DNA substrate. The dRP lyase reaction was initiated by adding the indicated amount of wild-type Pol β or the exon α Pol β variant and transferring the reaction mixture to 37 °C. Aliquots (4 µl each) were transferred at the indicated time intervals into tubes that contained 1 µl of freshly prepared 100 mM NaBH_4_. Reaction mixtures were shifted to 0–1 °C (on ice) and incubation was continued for 30 min. After incubation, the reaction was stopped by addition of 5 μl DNA gel-loading dye solution. The reaction mixtures were heated at 75 °C for 2 min, and the products were separated by electrophoresis in a 16% polyacrylamide gel, as above. Imaging and data analysis were performed by PhosphorImager and ImageQuant software. Data were fitted to a straight-line equation.

### NaBH_4_ reduction and cross-linking

NaBH_4_ reduction and crosslinking of wild-type Pol β or the exon α Pol β variant was performed with the 3′-FAM-labeled DNA substrate essentially as described previously^32,33^. Briefly, the reaction mixture (10 µl) contained 50 mM HEPES, pH 7.4, 1 mM EDTA, 2 mM dithiothreitol, 200 nM 3′-FAM-labeled duplex DNA, and the indicated amounts of wild-type Pol β or the exon α Pol β variant. For Schiff base reduction, 5 mM NaBH_4_ was used. The reaction mixture was incubated for 60 min on ice, followed by a 10 min incubation at 30 °C for NaBH_4_ reduction. After incubation, the reaction was terminated by addition of 10 µl SDS-PAGE gel-loading dye. Samples were heated for 5 min at 95 °C, and the protein-DNA cross-linked complexes were separated by using a Nu-PAGE Bis-Tris gel (10%) and MOPS running buffer system. A Typhoon PhosphorImager (GE HealthCare) was used for scanning the gels.

### *In vitro* BER assay

The BER assay was conducted in a final reaction mixture volume of 10 μl, as described previously^34^. Briefly, the repair reaction mixture, assembled on ice, contained 50 mM HEPES, pH 7.5, 20 mM KCl, 5 mM MgCl_2_, 0.5 mM EDTA, 2 mM DTT, 2 mM ATP, 5 μM [α-^32^P]dCTP (specific activity, 1 × 10^6^ dpm/pmol), and 250 nM DNA substrate (pretreated with 20 nM UDG), 10 nM APE1, 250 nM DNA ligase I, and wild-type Pol β or the exon α Pol β variant, as indicated. The repair reactions were then initiated by transferring reaction mixtures at 37 °C for 10 min. The reaction was stopped by addition of an equal volume of DNA gel loading buffer (95% formamide, 20 mM EDTA, 0.02% bromophenol blue, and 0.02% xylene cyanol) and incubated at 75 °C for 2 min. The reaction products were separated by electrophoresis in a 16% polyacrylamide gel containing 8 M urea, and the data were analyzed, as above.

## Results

### Detection of mRNA encoding the exon α Pol β variant in cancer cell lines

**Figure 1:**
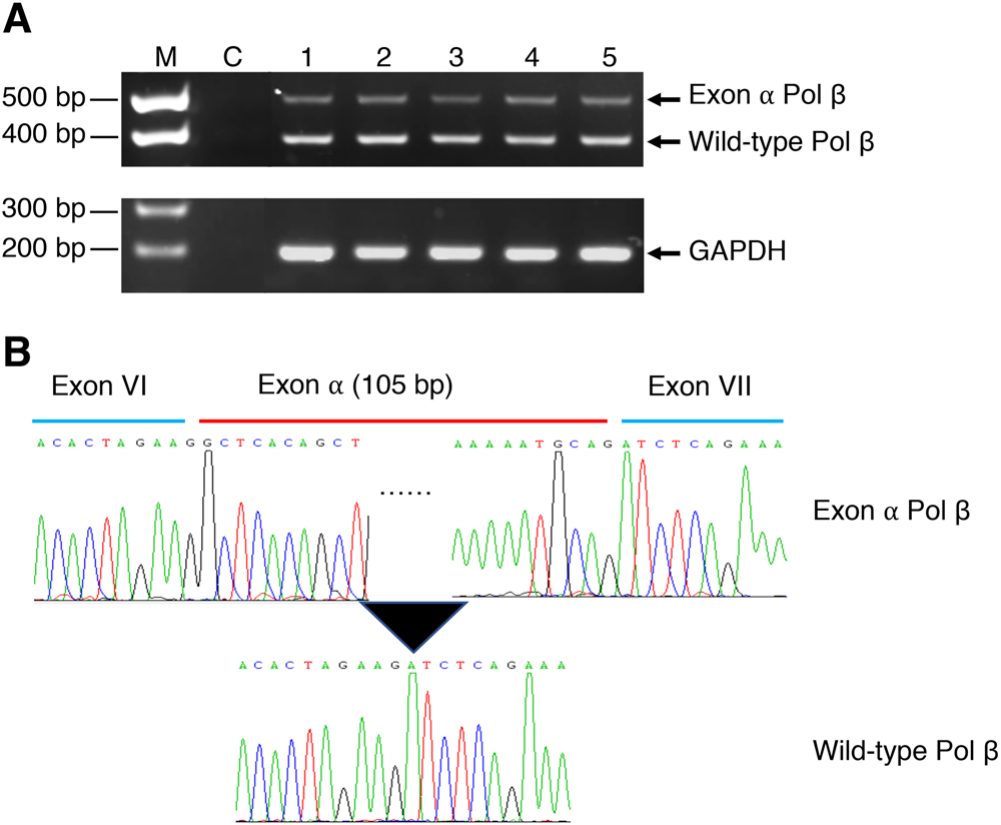
Detection of exon α Pol β in different cancer cell lines by RT-PCR. (**A**) Photograph of an ethidium bromide stained agarose gel illustrating RT-PCR products from different cell lines is shown. Total RNA was isolated using RNeasy kit and 1 μg RNA was used for the generation of cDNA template by SuperScript III First-Strand Synthesis System (Invitrogen). Two microliters of cDNA template from each cell line was used for the amplification of fragment from exon V to exon X. The lanes indicated by M, dsDNA markers; C, mock reaction mixture; Lanes 1 to 5 denote cell lines from the ATCC, SW480 (colon); 2, MCF7 (breast); 3, AGS (stomach); 4, A549 (lung); and 5, Y79 (eye), respectively. The sizes of DNA fragments generated by specific primers for wild-type Pol β and exon α Pol β were 363-bp and 468-bp, respectively. GAPDH gene product was used as reference. (**B**) Sequencing electropherogram of PCR products. RT-PCR products were gel-purified and used for sequencing of the region from exon V to exon X in the Pol β gene. Boundaries of exon VI, exon α, and exon VII are illustrated above the chromatograms. Black dotted line (……) indicates the omitted sequence of exon α. Detailed sequence information can be found in this here^27^.

To better understand the expression of the exon α Pol β variant in cancer cell lines, we used RT-PCR to analyze the mRNA expression level among several different types of cell lines. As shown in Fig. 1, RT-PCR products with exon α could be detected in all cancer cell lines studied, and the expression appeared to be at a similar level. The level of the mRNA containing exon α corresponded to approximately 1/10th the level of the normal Pol β mRNA (Fig. 1A). In each case, the insert of 105 nucleotides between exons VI and VII of Pol β gene was confirmed by DNA sequencing (Fig. 1B).

**Figure 2:**
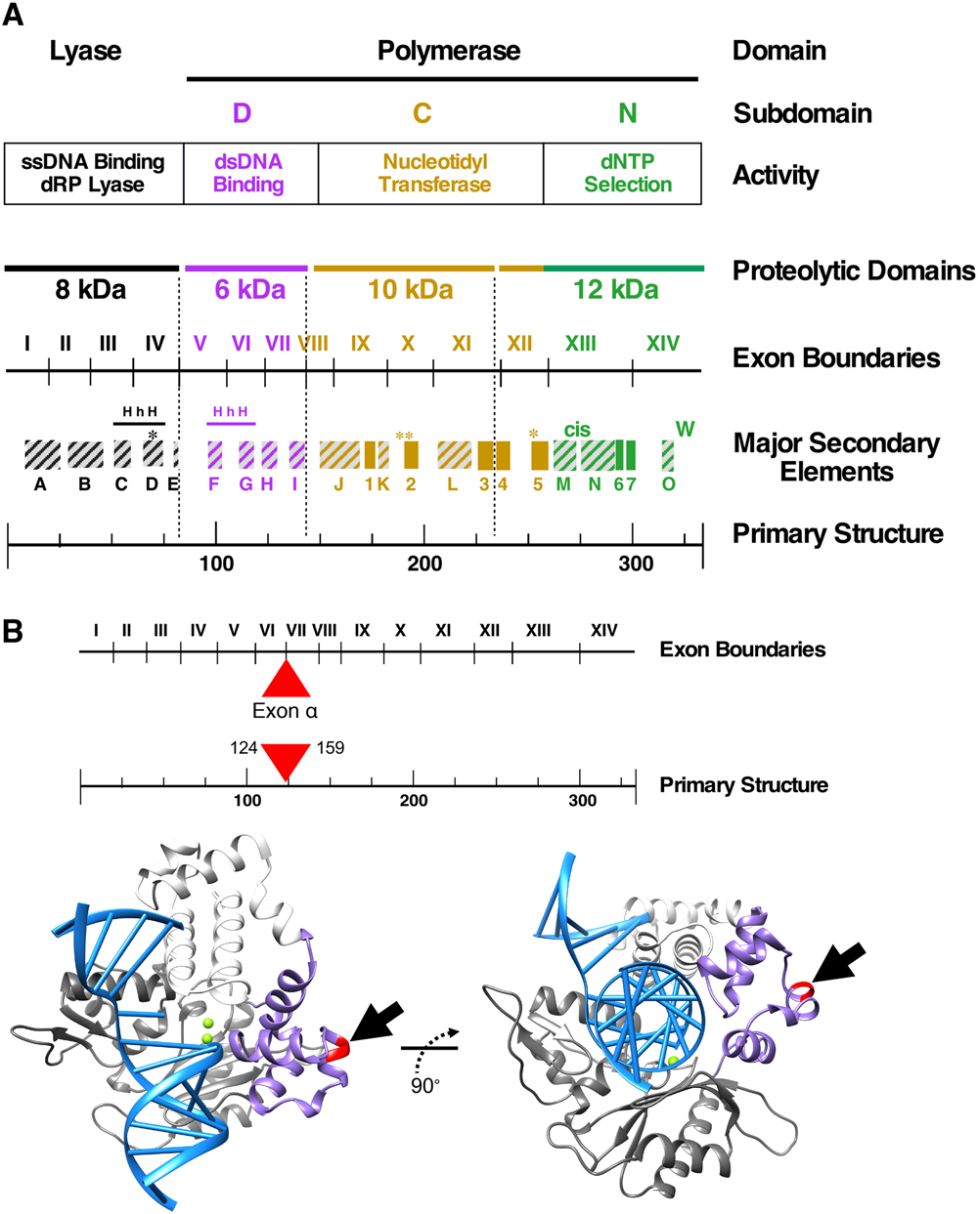
Structural organization of DNA polymerase β. (**A**) Schematics illustrating the primary structure, subdomains, proteolytic domains, exon boundaries and major secondary elements of DNA Pol β gene. Pol β has two major domains, lyase and polymerase domain. These domains are further subdivided into ssDNA binding dRP lyase, dsDNA binding, nucleotidyl transferase, and dNTP selection subdomains that can be generated by limited proteolysis. Pol β is composed of 14 exons that are indicated by I to XIV. The position of α-helices (boxes with stripes) and β-sheets (filled boxes) in the coding region of Pol β are indicated. Critical residues Lys72 in the lyase domain and Asp190, Asp192, and Asp256 in the polymerase domain are marked by asterisks (*). HhH refers to the helix-hairpin-helix motif observed in several DNA repair proteins. The relative positions of lone tryptophan (W) and *cis*-peptide (cis) bond are indicated. (**B**) Exon boundaries and primary structure of Pol β is as in panel A. The position of exon α insertion between exon VI and VII or between residues from 124 and 159 is indicated by a filled triangle (in red). An overview of Pol β bound to the duplex DNA helix image (left-hand side) and the same image of Pol β bound to duplex DNA helix (right-hand side) in which the template strand is bent 90°. The relative position of insertion of exon α in the dsDNA binding subdomain is illustrated by an arrow. Molecular images were produced with Chimera^48^ and MSMS^49^. This Figure was adopted from a review article by Beard and Wilson^8^.

The primary structure of the wild-type Pol β polypeptide, domains/subdomains organization, exon boundaries, and major secondary structure elements of the enzyme are shown in Fig. 2A. To provide a better understanding of the position of the 35 amino acid insert in the Pol β polypeptide, an overview of the structure of Pol β complexed with DNA is shown in Fig. 2B. The surface location of the exon α insert and its predicted secondary structure are illustrated in Fig. 2B.

### Evidence for expression of the exon α Pol β variant Pol β in MEFs

**Figure 3:**
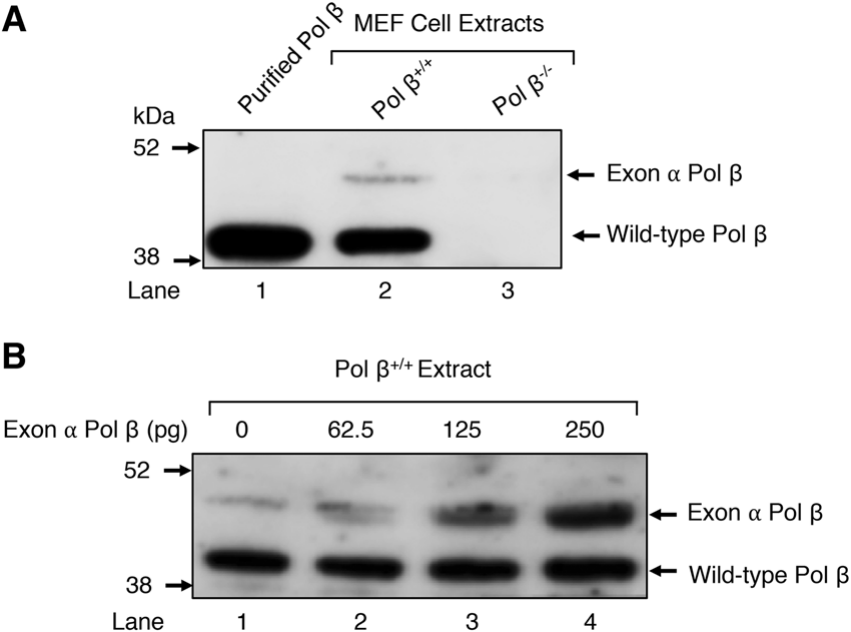
Detection of the exon α-containing Pol β variant Pol β expression in MEFs. (**A**) Image of an immunoblot illustrating expression of exon α Pol β in MEFs. Whole cell extract (20 μg each) from Pol β^+/+^ (lane 2) and Pol β^−/−^ (lane 3) MEF cells were separated by NuPAGE 4–12% Bis-Tris gel and transferred onto a nitrocellulose membrane. Purified wild-type Pol β (10 ng) in lane 1 served as reference. The immunoblot was developed with polyclonal antibody against the 8 kDa amino-terminal domain of Pol β (1:1000), as described under Materials and Method section. (**B**) Co-migration of purified exon α Pol β with endogenous exon α Pol β in the Pol β^+/+^ extract. Whole cell extract from Pol β^+/+^ MEF cells was supplemented either with 0 pg (lane 1), 62.5 pg (lane2), 125 pg (lane 3), or 250 pg (lane 4) of purified exon α Pol β and processed as in panel A. The relative positions of wild-type Pol β, exon α Pol β, 52 kDa and 38 kDa protein markers are indicated.

Having observed the exon α mRNA in mammalian cells, we wished to investigate the expression of the exon α Pol β variant at the protein level. Since the exon α insert is 105 nucleotides long, an ORF 35 amino acids longer than the wild-type Pol β ORF will result in a bigger size variant of Pol β. To examine whether the 42 kDa polypeptide is expressed in MEF cells, immunoblotting analysis of Pol β^+/+^ and Pol β^−/−^ MEFs extract was performed with an affinity purified polyclonal antibodies to the 8 kDa domain and the full-length 39 kDa Pol β. Interestingly, a higher molecular mass polypeptide, migrating slower than the wild-type Pol β polypeptide, was detected by the antibodies, as illustrated in Fig. 3A, lane 2. As expected, such an antibody reactive polypeptide was not observed in the Pol β^−/−^ extract (Fig. 3A, lane 3). To confirm the gel migration of the higher molecular mass polypeptide, we supplemented the Pol β^+/+^ extract with 0.125 ng and 0.25 ng (Fig. 3B, lanes 2 and 3) of the purified recombinant exon α Pol β variant (see below). Samples were then separated by SDS-PAGE and immunoblotting was performed as above. The results revealed that the recombinant exon α Pol β variant co-migrated in the gel with the endogenous larger Pol β polypeptide (Fig. 3B). These results indicated that the higher molecular mass polypeptide observed in the Pol β^+/+^ extract (Fig. 3A, lane 2), likely represented expression of the exon α-containing Pol β mRNA; the higher molecular mass polypeptide was a minor component compared with the wild-type Pol β polypeptide.

### Expression of recombinant exon α Pol β in *E. coli*

**Figure 4:**
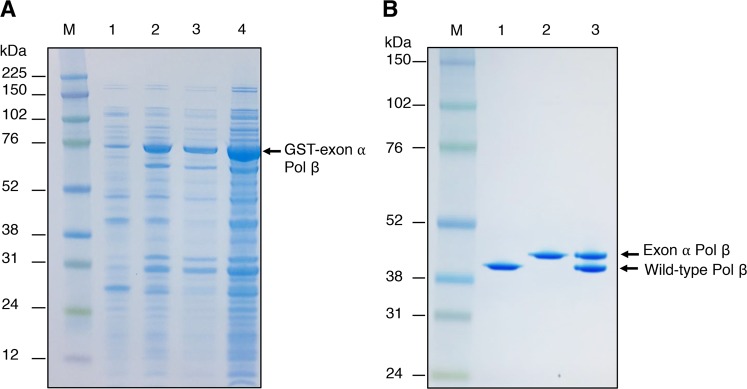
Expression and purification of GST-tagged exon α Pol β. The details of expression and purification of GST-tagged exon α Pol β are described under Materials and Methods section. Images of Coomassie blue-stained gels are shown. (**A**) Lane 1, total cell lysate of *E. coli* carrying exon α Pol β gene expression vector before IPTG induction; lane 2, total cell lysate of *E. coli* after IPTG induction; lane 3, the supernatant fraction after sonication and ultra-centrifugation; and lane 4, the pellet fraction after sonication and ultracentrifugation. The protein markers are in lane M. The positions of GST-exon α Pol β and the protein markers are indicated. (**B**) The GST tag was removed during purification protocol as detailed in Materials and Methods. The final preparations of purified wild-type Pol β and exon α Pol β are shown. An aliquot of 2 μg each of wild-type Pol β (lane 1), exon α Pol β (lane 2), and a mixture of wild-type Pol β and exon α Pol β (lane 3) was separated by12% SDS PAGE gel. The protein markers are in lane M. The positions of wild-type Pol β and exon α Pol β are indicated.

Our previous efforts toward recombinant expression of a soluble form of exon α Pol β in *E. coli* were not successful; thus, using a heat-inducible system, the 42 kDa polypeptide was expressed, but remained insoluble in the pellet fraction^27^. To overcome this problem, we tagged the exon α-containing human Pol β cDNA with Glutathione S-transferase (GST) in the pGEX4T vector. We then expressed the corresponding protein in *E. coli* (BL21(DE3)-CodonPlus-RP cells, as described under Materials and Methods. Under these conditions a substantial amount of GST-tagged exon α Pol β was recovered in the supernatant fraction (Fig. 4A, lane 3). Therefore, we purified both wild-type Pol β and exon α Pol β from the supernatant fraction using GST Sephasrose 4B and Resource S columns. Since the insertion of 35 amino acids from exon α into the wild-type Pol β protein resulted in a lower protein isoelectric point as compared to wild-type Pol β, MES buffer at pH 6.5 was used in the final step of purification by Resource S column chromatography. The electrophoretic mobilities of purified wild-type Pol β (39 kDa), exon α Pol β (42 kDa), and a mixture of both of these forms of Pol β, were compared in Fig. 4B, lanes 7–9.

### Effect of the exon α insertion on DNA repair properties of Pol β

#### DNA binding

**Figure 5:**
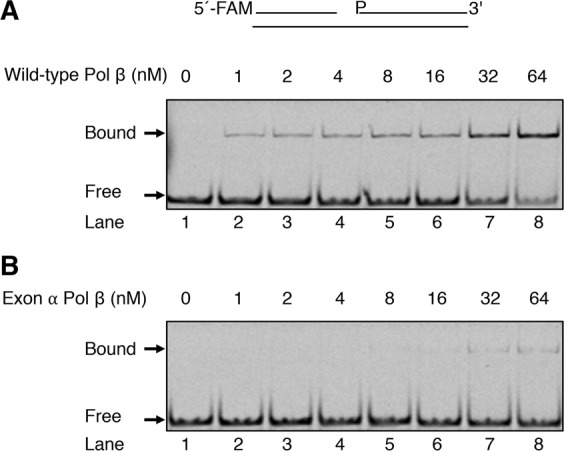
DNA binding affinity of wild-type Pol β and exon α Pol β to 1-nt gapped DNA. Representative fluorescent images of DNA binding affinity of wild-type Pol β (**A**) and exon α Pol β (**B**). Binding reaction was performed as described under Materials and Methods section. Different protein concentrations (0–64 nM) of wild-type Pol β or exon α Pol β were mixed with 5′-FAM labeled 1-nt gapped DNA and incubated for 5 min at room temperature. After incubation, the enzyme-DNA complexes were separated from free DNA by non-denaturing polyacrylamide gel electrophoresis. Schematic representation of the substrate is shown on top of the image. The positions of bound (protein-DNA complexes) and free DNA are indicated. A representative phosphorimage of three experiments is illustrated.

As illustrated in Figs 1 and 2, exons VI and VII are located in the 6 kDa dsDNA binding region of Pol β^8,27^. We reasoned that insertion of exon α between exons VI and VII might hinder/alter the dsDNA binding affinity of exon α Pol β, as compared to wild-type Pol β. A qualitative assessment of DNA binding of wild-type Pol β and exon α Pol β was conducted utilizing a 5′-FAM-labeled DNA containing a 1-nucleotide gap between positions 15 and 17 and a native gel electrophoresis technique^35^. The exon α Pol β showed reduced binding affinity for the gapped DNA substrate compared with that of wild-type Pol β (Fig. 5). In the case of wild-type Pol β, at the 32 nM level of enzyme, approximately 50% of the DNA was found in an enzyme-DNA complex, whereas in the case of exon α Pol β at the same level of enzyme only a negligible amount of enzyme-DNA complex was observed. Thus, these results suggested that the presence of exon α in the dsDNA binding region of the enzyme hindered the DNA binding capacity of Pol β.

### DNA synthesis

**Figure 6:**
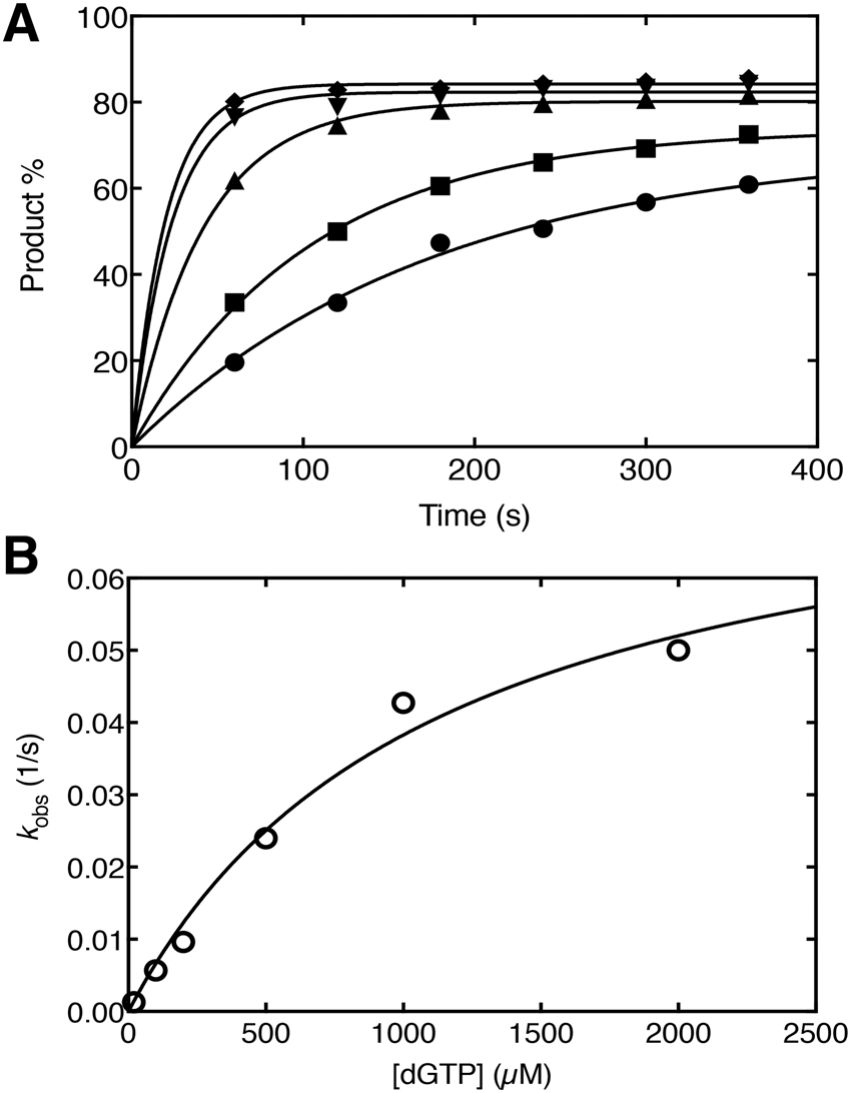
Kinetic measurements of gap-filling DNA synthesis of exon α Pol β. (**A**) The polymerase gap filling activity assay was performed under single-turnover reaction conditions as a function of dGTP concentration with 100 μM (●), 200 μM (■), 500 μM (▲), 1000 μM (▼), and 2000 μM (♦). Time courses were fit to a single exponential. (**B**) A secondary plot of the dGTP concentration dependence of the observed first-order rate constants (*k*_obs_). These data were fit to the Michaelis equation by nonlinear least-squares methods to derive *k*_pol_ and *K*_d_ (Table 1).

We examined DNA synthesis using reaction conditions appropriate for single-turnover analyses, *i.e*., enzyme excess over DNA (ratio of enzyme/DNA = 10). This enabled measurement of insertion (apparent *k*_*pol*_) and dNTP binding (*K*_d,dNTP_) parameters. The exon α Pol β apparent rate constant for insertion, *k*_pol_, and affinity for incoming nucleotide, *K*_*d*_, were 0.08 s^−1^ and 1118 μM, respectively (Fig. 6). In contrast, *k*_pol_ and *K*_*d*_ values for wild-type Pol β were 14 s^−1^ and 1 μM (Table 1)^30^. These results indicated the nucleotide insertion activity of exon α Pol β was severely impaired.

**Table 1:** Single-turnover kinetic parameters of the gap-filling activity of wild-type Pol β and exon α Pol β.

Measurements	wild-type Pol β*	α-isoform Pol β	Ratio
*k*_pol_ (1/s)	14	0.1	140
*K*_d_ (μM)	1	1118	8.9 × 10^−4^

*Data were taken from Sucato *et al*.^30^.

### dRP lyase activity

**Figure 7:**
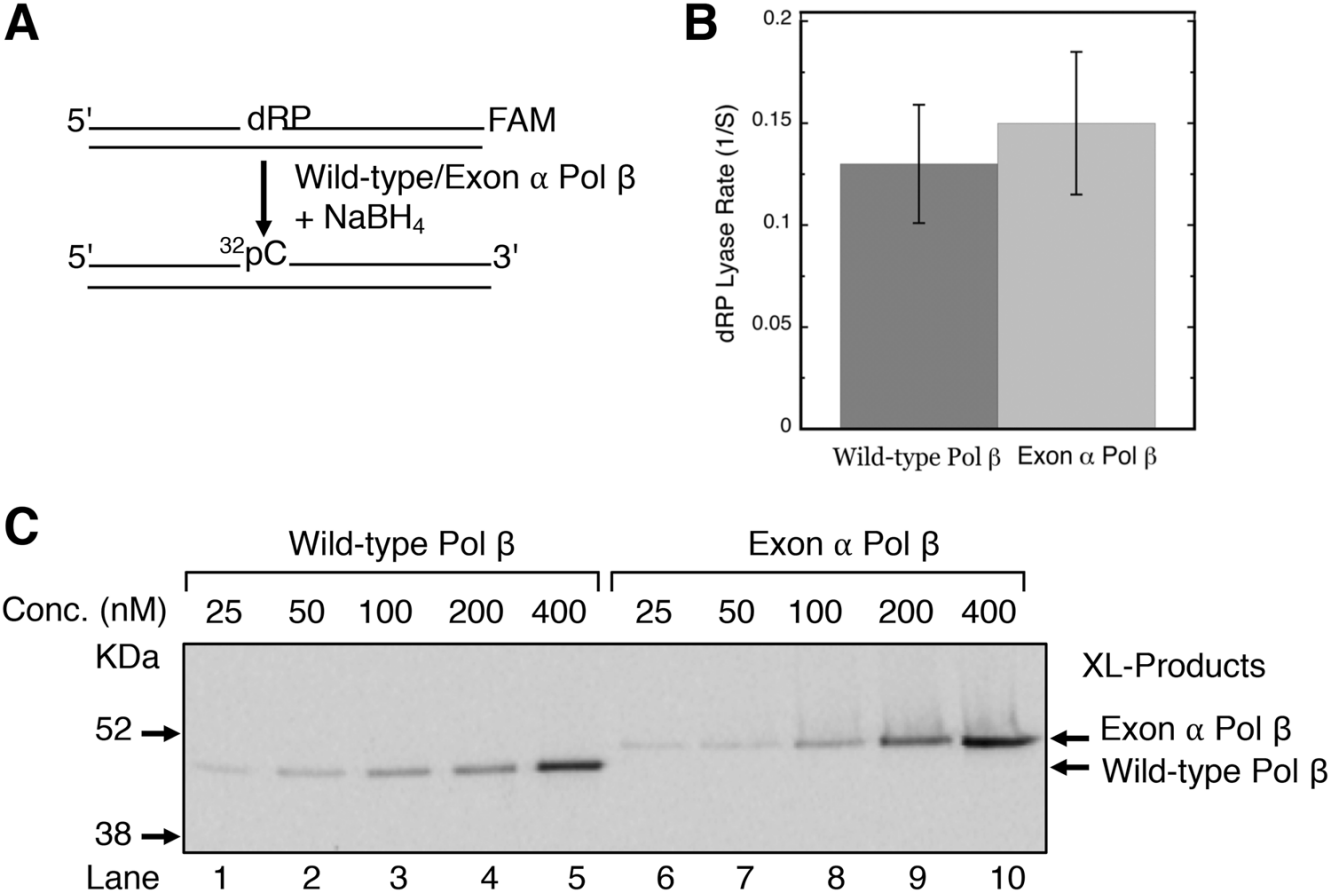
Kinetic measurements of dRP lyase and NaBH4 cross-linking of wild-type Pol β and exon α Pol β. (**A**) Schematic representation of the UDG pretreated DNA substrate used for dRP lyase rate determination and covalent cross-linking by NaBH_4_ reduction. (**B**) Release of dRP from incised AP site-containing DNA substrate was examined under steady-state reaction conditions as a function of incubation time. The reactions were performed as describes under “Material and Methods” with 20 nM wild-type Pol β or exon α Pol β and 200 nM DNA. Time course data were analyzed using ImageQuant software and fitted to a straight-line equation. An average dRP lyase rates of three independent experiments was plotted. (**C**) Covalent cross-linking of wild-type Pol β or exon α Pol β. Incised AP site-containing DNA substrate (200 nM) was incubated in a reaction mixture with 25 to 400 nM wild-type Pol β (lanes 1 to 5) or exon α Pol β (lanes 6 to10), and 5 mM NaBH_4_. The reaction mixtures were incubated on ice for 1 h followed by incubation at room temperature for 10 min. Covalently cross-linked DNA-protein products were separated by 10% NuPAGE Bis-Tris gel, and the gel was scanned on a PhosphorImager. The positions of cross-linked products are indicated. Relative positions of protein markers are indicated on the left side of the phosphorimage. A representative phosphorimage of two experiments is illustrated.

The dRP lyase activity of Pol β is contributed by the amino terminal 8 kDa domain’s ssDNA binding region, and the region is capable of binding independently to the 5′-phosphate in a gapped DNA substrate (see Fig. 2A). The 8 kDa domain is distal to the exon α insertion, and we were interested to know whether the dRP lyase activity of Pol β is altered by the exon α insertion. To compare kinetic parameters of dRP lyase activity by wild-type Pol β and exon α Pol β, we used a duplex DNA substrate (34-bp) containing a 5′-dRP group at an internal nick and the 3′-FAM-label at the 3′-end of DNA (Fig. 7A). The resulting DNA substrate with a 5′-dRP group at a nick and FAM label at the 3′-terminus was incubated with wild-type Pol β or exon α Pol β. Removal of the dRP group from the FAM labeled strand was monitored by electrophoresis in a denaturing polyacrylamide gel (Fig. S1). A control reaction without enzyme was incubated under similar conditions to account for a non-enzymatic background in the reaction product, as the dRP group in the substrate is heat labile. Both enzymes released the dRP group from the substrate in a time-dependent manner (Fig. S1). The rates were determined under steady-state kinetics conditions (i.e., ratio of DNA/enzyme = 10); the rates for wild-type Pol β and exon α Pol β were similar (Fig. 7B), indicating the presence of exon α had no impact on the dRP lyase activity.

### NaBH_4_ cross-linking

**Figure 8:**
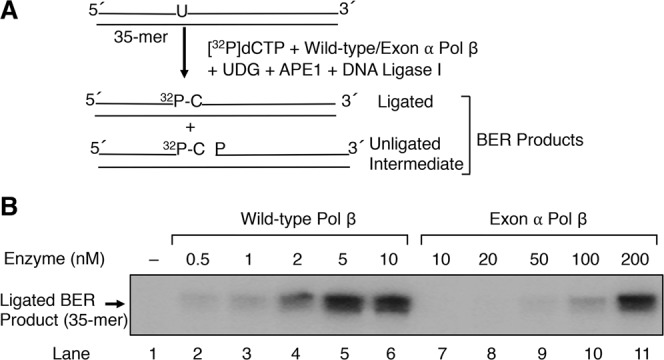
*In vitro* BER activity of wild-type Pol β and exon α Pol β. (**A**) Schematic representation of the *in vitro* BER substrate (35-base pair duplex DNA containing uracil opposite guanine) and the expected BER products after replacement of uracil with [^32^P]dCMP (ligated 35-mer and 16-mer unligated intermediate). (**B**) Phosphorimage of a denaturing PAGE illustrating the *in vitro* BER activities of wild-type Pol β and exon α Pol β. BER activity of wild-type Pol β or exon α Pol β was evaluated using an uracil-containing BER substrate by measuring incorporation of [^32^P]dCMP as a function of protein concentrations. Reaction conditions and product analysis were as described under “Materials and Methods.” A 35-bp oligonucleotide duplex DNA (250 nM), pretreated with UDG, was used in a reaction mixture with 0.5 to 10 nM wild-type Pol β or 10 to 200 nM exon α Pol β, 10 nM APE1, and 250 nM DNA ligase I. The reaction products were separated by 16% denaturing PAGE. In the figure only ligated BER product (35-mer) is shown. The migration position of ligated BER product is indicated. A representative phosphorimage of three experiments is illustrated.

To further verify and illustrate that the dRP lyase activity of exon α Pol β was intrinsic to the ~42 kDa polypeptide and similar to wild-type Pol β, we used a NaBH_4_ cross-linking technique^32,36^. In this cross-linking assay, the active site primary amine of the enzyme attacks the electrophilic C1′ atom of the 5′-dRP sugar yielding a Schiff base intermediate, which is converted to a stable covalent enzyme-DNA complex upon NaBH_4_ reduction. When wild-type Pol β and exon α Pol β were incubated with 5′-dRP-containing DNA and NaBH_4_, protein-DNA complexes of ~45 kDa and ~48 kDa, respectively, were observed (Fig. 8B, lanes 1–5 and lanes 6–10). The electrophoretic mobilities of these ~45 kDa and ~48 kDa protein-DNA complexes were consistent of the molecular masses of wild-type Pol β and exon α Pol β plus 18-mer FAM-labeled DNA, respectively (Fig. 7C). The protein-DNA complex formation increased with increased enzyme concentration to approximately the same level in both cases, further suggesting that the proteins had similar dRP lyase activities.

### *In vitro* base excision repair activity

Next, in view of the gap-filling deficiency described above, we tested whether exon α Pol β was capable of supporting any level of *in vitro* BER. Reaction mixtures containing a 35-bp DNA substrate with uracil opposite guanine were incubated with purified BER enzymes UDG, APE1, DNA ligase I, and wild-type Pol β or exon α Pol β. Reactions were initiated by the addition of Mg^2+^ and [α-^32^P]dCTP, and repair activity was evaluated by measuring incorporation of [^32^P]dCMP as a function of protein concentrations. The results shown in Fig. 8 indicated that while the uracil lesion in DNA was strongly repaired by wild-type Pol β, the repair capacity of exon α Pol β was severely compromised, indicating again the nucleotide insertion activity of exon α-containing Pol β was impaired and as a result functional BER was sharply reduced.

## Discussion

It has been demonstrated that the diversity of splice variants in human Pol β mRNA is remarkably large^25,26,37^. Our previous report indicated that at least eight variant isoforms could be detected in normal and cancer cell lines although most of them would generate a premature termination codon and truncated polypeptides^27^. Recently, Skandalis *et al*.^37^ reported over 30 splice variants in normal and emetine treated human cells. In that report, the exon α-containing Pol β variant was detected and represented around 5~9% of Pol β transcripts, which is in accord with the data in our current and previous studies (Fig. 1). Our current data revealed that all the cancer cell lines tested expressed a relatively small and similar amount of the exon α-containing Pol β variant mRNA. From these data, we inferred that exon α Pol β was common in different types of cancer cell lines.

Since the discovery of exon α Pol β, no investigation of functional implications concerning this variant had been reported, likely due to its insoluble properties in recombinant expression systems^27^. Because the Glutathione S-Transferase tag approach is known to significantly enhance solubility of fusion protein in *E. coli*^38^, we fused the coding region of the exon α-containing Pol β cDNA with the GST tag and then evaluated overexpression; although ~50% of recombinant exon α Pol β was found to be soluble (Fig. 4), this provided ample protein to achieve purification. As the insertion site of exon α is located between exons VI and VII falling within the dsDNA-binding region of Pol β (see Figs 1 and 2), we speculated that the insertion may have an impact on various Pol β activities^1,8,27^. However, the insertion is on the surface of the enzyme and distal to the enzyme’s active sites (Fig. 2B). As shown in Fig. 5, the gapped DNA binding capacity of α-exon Pol β appeared to be reduced significantly, and the insertion gap-filling and BER activities of exon α Pol β were approximately 1/1000 of those of the wild-type enzyme (Fig. 6 and Table 1).

Many reports have described genomic alterations in Pol β in various types of human cancer, including colon, gastric, prostate, lung, breast and esophageal^39–42^. Many tumors examined to date may express Pol β variant proteins that are predicted to have a single amino acid substitution. It has been reported that some of these variants, such as E295K^43,44^, Y265C^45^, and R152C^46^, are associated with reduced DNA polymerase activity, potentially leading to genome instability and/or cellular transformation. Besides these examples, deletions of exon II or exon XI and a mRNA with a 107-nucleotide insertion between exon IX and exon X have been suggested to be linked to the development of colon cancer and Werner’s syndrome, respectively, by others^25,26^.

In the current study, we found that the insertion of exon α into the junction site of exon VI and exon VII resulted in a protein with strongly reduced DNA binding affinity and DNA polymerase activity. This Pol β variant exhibited only a minor fraction of the DNA repair capacity compared to that of the wild-type enzyme. To investigate the biological implication of this Pol β variant, we explored the protein expression pattern of exon α Pol β in cancer cell lines. However, the exon α Pol β protein was below the level of detection in the human cell lines tested (data not shown), although we could detect exon α Pol β in mouse MEFs (Fig. 3). Further, we expressed the recombinant variant protein in Pol β null mouse embryonic fibroblasts. Yet, the expression level of the Pol β variant was low, compared with that of wild-type Pol β (data not shown). These data indicated the 35 amino acid exon α insertion decreased expression or accelerated degradation of the variant enzyme in these cells. In spite of this, another study found that the expressed transcript of exon α Pol β was stable in normal and cancer cell lines, even after treatment with the translation blocking reagent emetine or under oxidative stress conditions^37,47^. More work is need to understand the biological significance of exon α Pol β.

In conclusion, using a GST-fusion recombinant expression system, we expressed and purified the exon α-containing Pol β variant. Biochemical characterization of the variant enzyme revealed that the insertion of the exon α into the coding region of Pol β diminishes the DNA synthesis activity of the variant enzyme, but not the dRP lyase activity.

## Supplementary information

The Pol β variant containing exon α is deficient in DNA polymerase but has full dRP lyase activity

## Data Availability

All data generated or analyzed during the current study are included.
