# Supplementary material for: The Pol β variant containing exon α is deficient in DNA polymerase but has full dRP lyase activity
Source: Sci Rep. 2019 Jul 9;9:9928. doi: 10.1038/s41598-019-45846-0 (PMC6616571; doi:10.1038/s41598-019-45846-0)
Supplement: Supplementary file 1 — The Pol β variant containing exon α is deficient in DNA polymerase but has full dRP lyase activity [file 41598_2019_45846_MOESM1_ESM.pdf]

Supplementary Information

The Pol β variant containing exon α is deficient in DNA polymerase but has full dRP lyase activity

Da-Peng Dai [a,b], Rajendra Prasad [a], Phyllis R. Strauss [a,c], Samuel H. Wilson [a,*]

[a] Genome Integrity and Structural Biology Laboratory, National Institutes of Health, NIEHS, 111 T.W. Alexander Drive, P.O. Box 12233, Research Triangle Park, NC 27709, USA
[b] Present address: The MOH Key Laboratory of Geriatrics, Beijing Hospital, National Center of Gerontology, Beijing 100730, P. R. China

[c] Present address: Biology Department, Northeastern University, 360 Huntington Avenue, Boston, MA 02115, USA

Running title: Characterization of exon α-containing isoform of Polβ

*To whom correspondence should be addressed: Genome Integrity and Structural Biology Laboratory, National Institutes of Health, NIEHS, 111 T. W. Alexander Dr., P. O. Box 12233 MD F1-12, Research Triangle Park, NC 27709.
Tel.: 919-541-4701;
Fax: 919-541-4724;
E-mail: wilson5@niehs.nih.gov.

Figure S1. dRp lyase activity of of wild-type Pol β and exon α Pol β. A. schematic representation of the UDG pretreated DNA substrate used for dRP lyase rate measurements. B. release of dRP from incised AP site-containing DNA substrate was examined under steady-state reaction conditions as a function of incubation time. The reactions were performed as describes under "Material and Methods" either with wild-type Pol β (lanes 6-10), or with exon α Pol β (lanes 11-15). Reactions in lanes 1-5 represent incubation without enzyme. The reaction products were separated by 16% denaturing PAGE. The migrations position of substrate and product are indicated. A representative phosphorimage of three experiments is illustrated.

A.

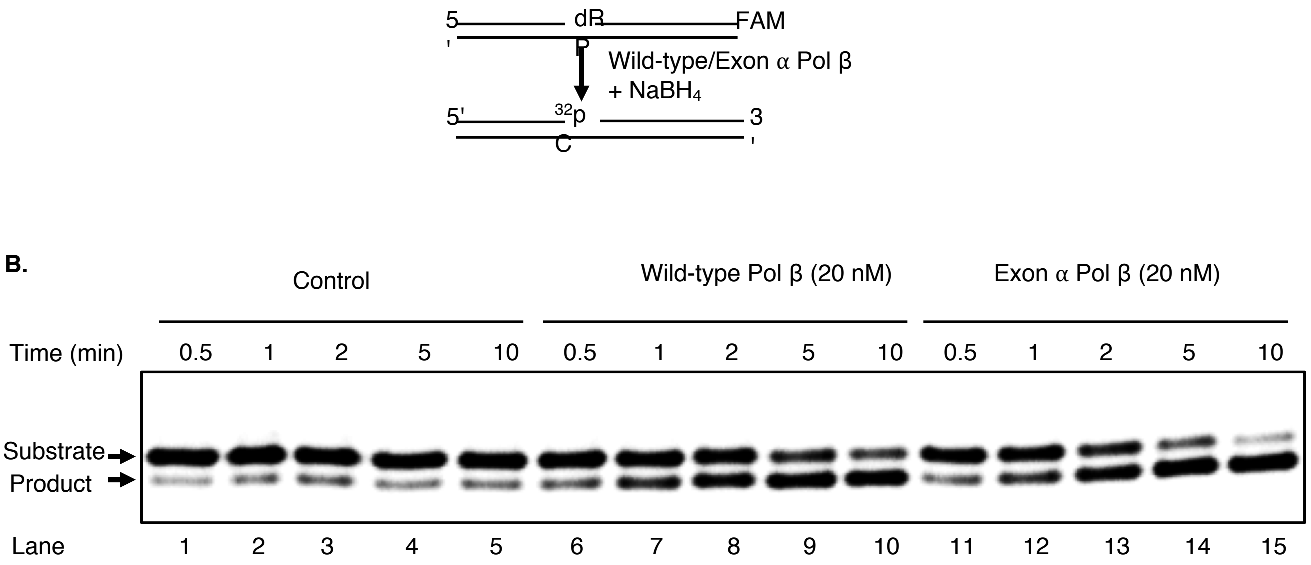

Supplementary Figure Figure S1

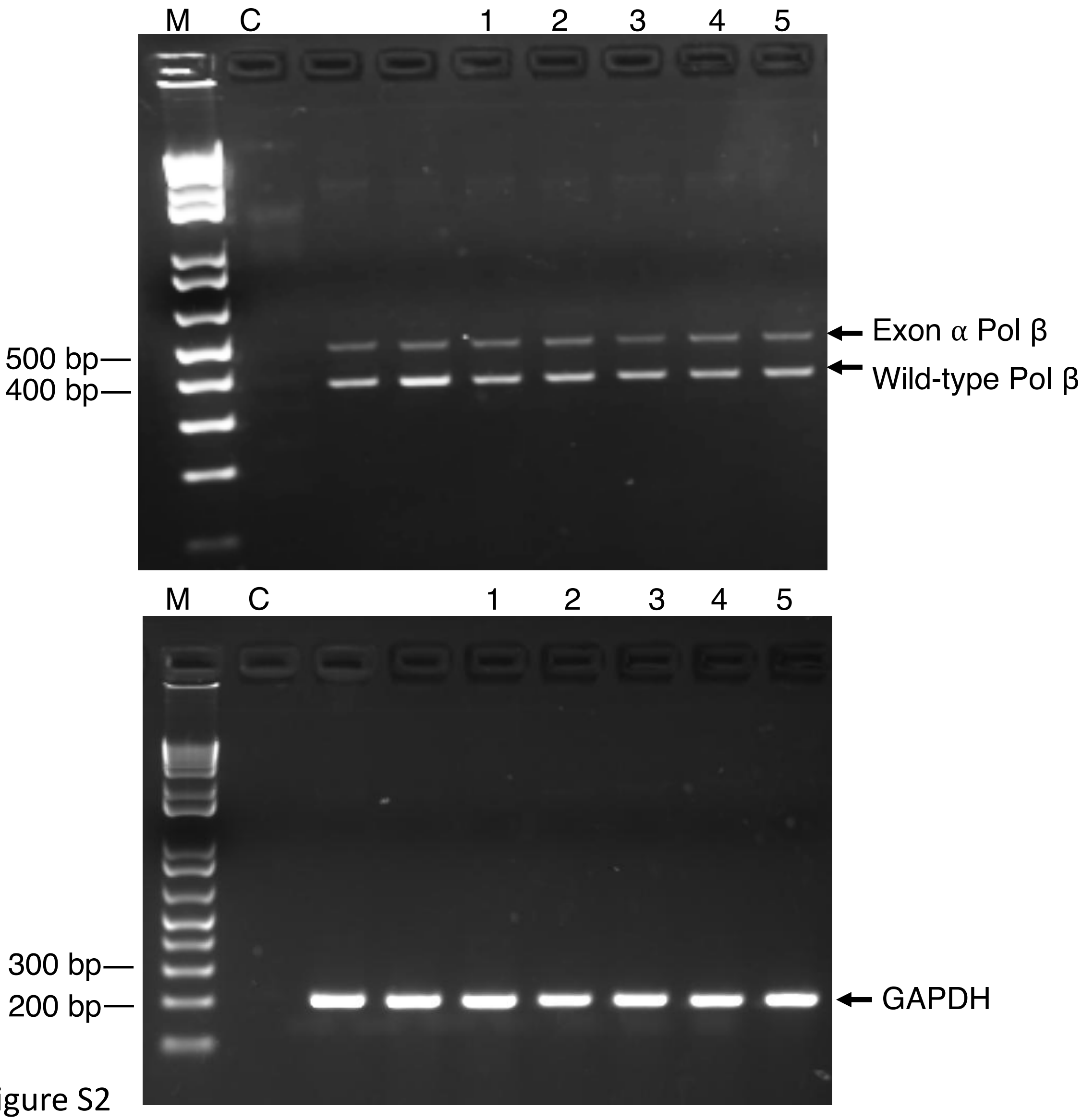

Supplementary Figure S2

Full agarose DNA gels for the main text Figure 1

Lanes denote M, DNA markers; C, mock reaction mixture; 1, SW (colon); 2, MCF7 (breast); 3, AGS (stomach); 4, A459 (lung); and 5, Y79 (eye), respectively, and were used for the main text Figure 1.

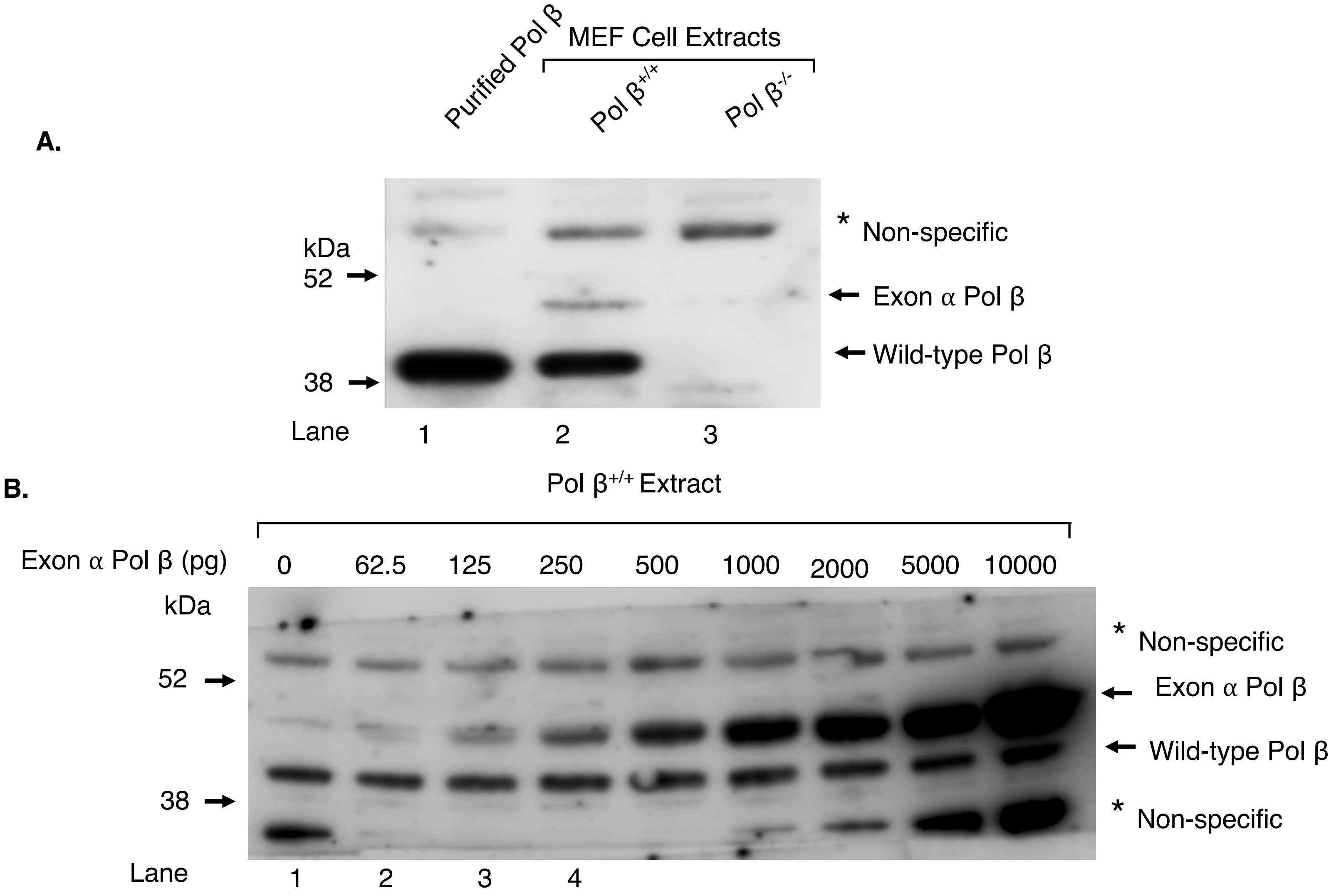

Supplementary Figure S3

Full gel Immunoblots for the main text Figure 3

A. Immunoblot for Figure 3A, * non-specific protein reacting to the antibodies.
B. Immunoblot for Figure 3B, in the main text figure 3 only lanes 1-4 were used.

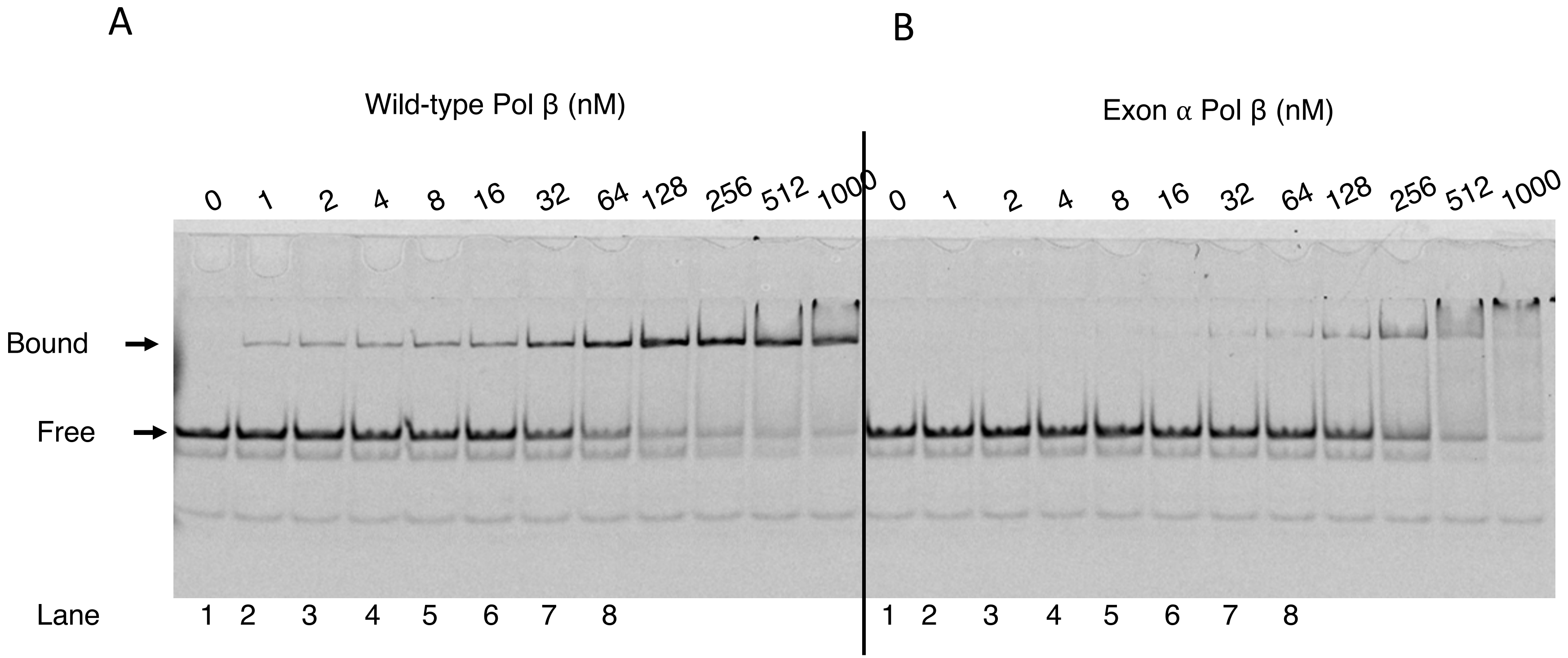

Supplementary Figure S4

Full gel image for the text Figure 5 and in both panels lanes 1 to 8 were used.

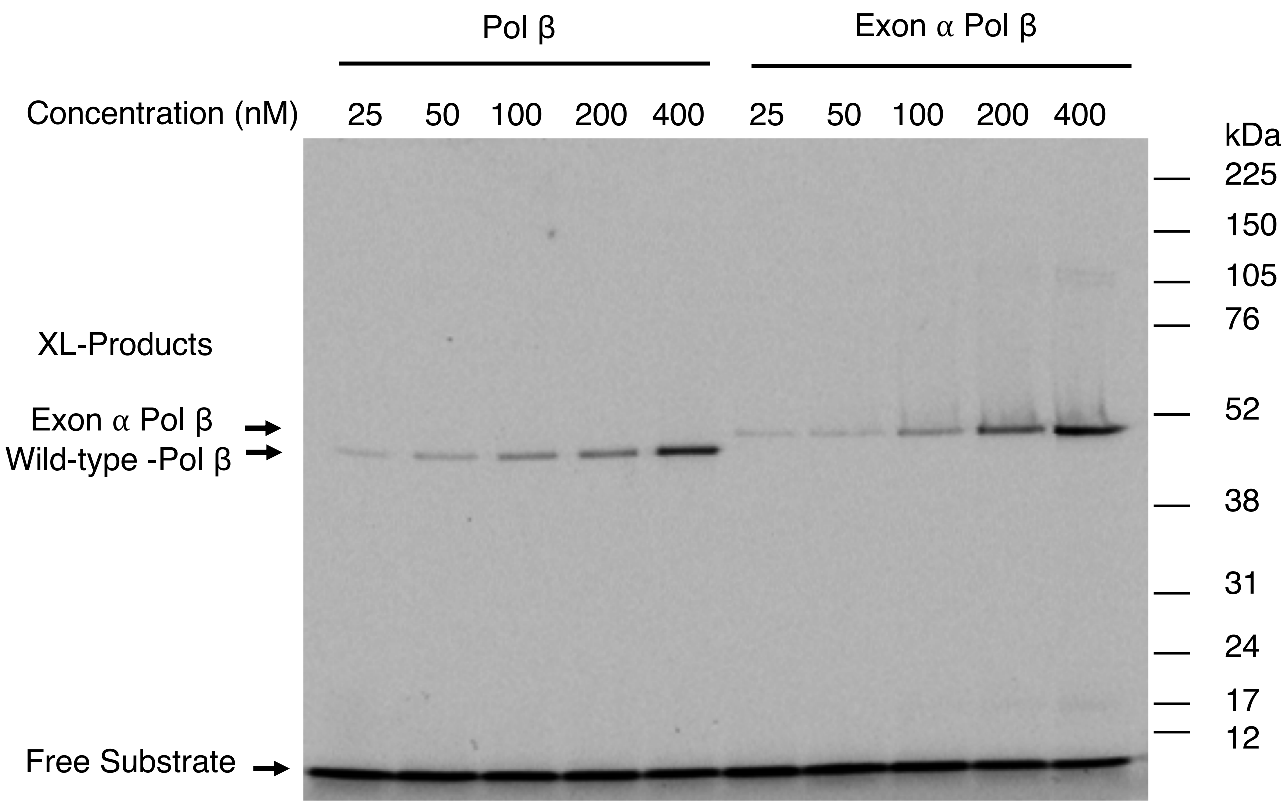

Supplementary Figure S5

Full SDS-PAGE gel phosphorimage for the text Figure 7C.

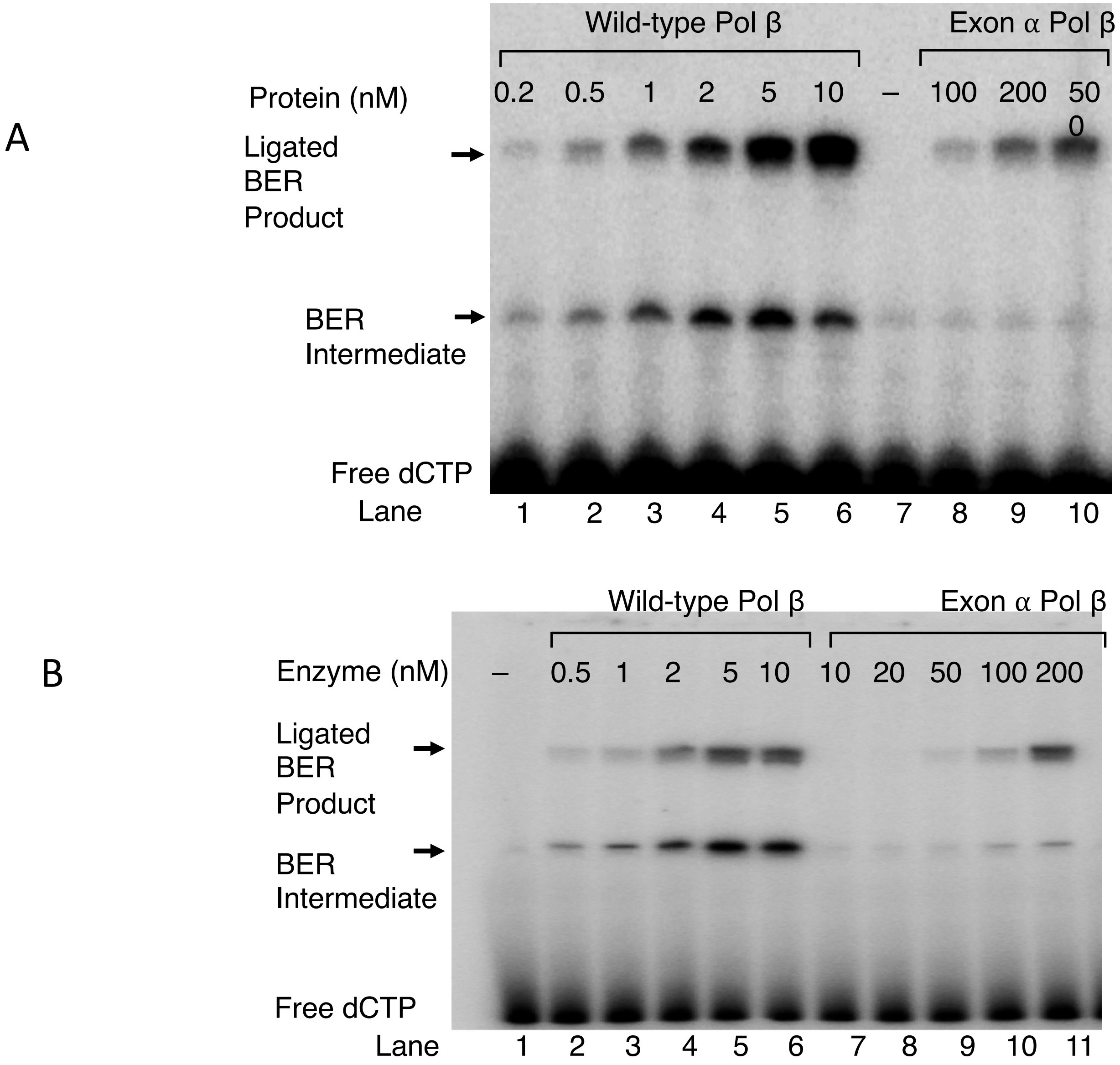

Supplementary Figure S6

Full gel phosphorimage for the text Figure 8 B and C.
